# Long-term outcome and predictors of neurological recovery in cervical spinal cord injury: a population-based cohort study

**DOI:** 10.1038/s41598-024-71983-2

**Published:** 2024-09-09

**Authors:** Vasilios Stenimahitis, Maria Gharios, Alexander Fletcher-Sandersjöö, Victor Gabriel El-Hajj, Aman Singh, Ali Buwaider, Magnus Andersson, Paul Gerdhem, Claes Hultling, Adrian Elmi-Terander, Erik Edström

**Affiliations:** 1 Department of Clinical Neuroscience, Karolinska Institutet, Stockholm, Sweden; 2 Department of Rehabilitation, Furuhöjden Rehab Hospital, Täby, Sweden; 3 Department of Surgical Sciences, Uppsala University, Uppsala, Sweden; 4 Department of Neurology, Uppsala University, Uppsala, Sweden; 5 Department of Orthopedics and Hand Surgery, Uppsala University Hospital, Uppsala, Sweden; 6 Department of Neurobiology, Care Sciences and Society, Karolinska Institutet, Stockholm, Sweden; 7 Capio Spine Center Stockholm, Löwenströmska Hospital, Stockholm, Sweden; 8 Department of Medical Sciences, Örebro University, Örebro, Sweden

**Keywords:** Spinal cord injury, Ambulation, American spinal injury association impairment scale, Neurological outcome, Predictors, Neurological disorders, Spinal cord diseases

## Abstract

This retrospective study analyzed prognostic factors for neurological improvement and ambulation in 194 adult patients (≥ 15 years) with traumatic cervical spinal cord injuries treated at the neurological SCI unit (SCIU) at the Karolinska University Hospital Stockholm, Sweden, between 2010 and 2020. The primary outcome was American spinal injury association impairment scale (AIS) improvement, with secondary focus on ambulation restoration. Results showed 41% experienced AIS improvement, with 51% regaining ambulation over a median follow-up of 3.7 years. Significant AIS improvement (p < 0.001) and reduced bladder/bowel dysfunction (p < 0.001) were noted. Multivariable analysis identified initial AIS C-D (< 0.001), central cord syndrome (p = 0.016), and C0–C3 injury (p = 0.017) as positive AIS improvement predictors, while lower extremity motor score (LEMS) (p < 0.001) and longer ICU stays (p < 0.001) were negative predictors. Patients with initial AIS C-D (p < 0.001) and higher LEMS (p < 0.001) were more likely to regain ambulation. Finally, older age was a negative prognostic factor (p = 0.003). In conclusion, initial injury severity significantly predicted neurological improvement and ambulation. Recovery was observed even in severe cases, emphasizing the importance of tailored rehabilitation for improved outcomes.

## Introduction

Spinal cord injury (SCI) encompasses a spectrum of trauma-induced impairments to the spinal cord, affecting sensory, motor, and autonomic functions^1,2^. Each year, SCI affects approximately 250,000 to 500,000 people globally^3^, with a prevalence that continues to grow^4^. While incidence rates demonstrate regional variance^5^, falls and road traffic accidents remain the primary cause^4,6^. However, the age profile of SCI victims is changing—while traditionally affecting younger adults, there is now a rising prevalence among the elderly due to falls^7^.

SCI induces a spectrum of symptoms that vary with the injury’s level and region^1^. These can manifest as deficits in sensory perception, motor function, and critical autonomic functions, including respiration and cardiovascular control. The cervical spine, vulnerable due to its flexibility and structure^8^, is susceptible to traumatic injuries of debilitating character. The American spinal injury association impairment scale (AIS) provides a classification system, ranging from complete injuries with no preserved motor or sensory functions (grade A) to normal function (grade E). Incomplete SCIs maintain some neural pathways, permitting limited sensory and motor function. The repercussions of SCI extend beyond physical health, encompassing significant social and economic challenges and leading to reduced quality of life^9,10^. Moreover, life expectancy is adversely affected^11^, with respiratory complications and septicemia as predominant causes of mortality post-SCI^7^.

A critical aspect of early SCI management involves predicting the patient’s potential for neurological recovery. Data indicate that a considerable proportion of patients regain a measure of function, predominantly within the first 3 months, although modest improvements can manifest up to 18 months after the injury^12^. The identification of predictors of neurological recovery would enable clinicians to help patients make well-founded decisions regarding rehabilitation strategies, financial considerations, and the establishment of achievable goals.

Considering the above, the aim of this study was to delineate the temporal profile and identify predictors of neurological recovery following traumatic cervical SCI.

## Methods

### Study design and setting

This was a retrospective single center observational cohort study that included all adults (≥ 15 years) with a cervical SCI who underwent rehabilitation at the neurological SCI unit (SCIU) at the Karolinska University Hospital, Stockholm, Sweden, between 2010 and 2020. The study hospital is a publicly funded and owned tertiary care center serving a region of roughly 2.3 million inhabitants, and the only SCIU in the region. Data were extracted from the patients’ electronic charts using the health record software TakeCare (CompuGroup Medical Sweden AB, Farsta, Sweden). The study was performed in accordance with the Declaration of Helsinki and all other relevant ethical guidelines. The National “Swedish Ethical Review Authority” approved the study (Dnr: 2020-02086) and waived the need for informed consent, as per the Swedish law on retrospective research. The study adhered to the strengthening the reporting of observational studies in epidemiology (STROBE) guidelines.

#### Variables and outcomes

Statistical outcome analyses were performed comparing admission data and last available follow-up data. In addition, descriptive data from 3 to 5 years follow-ups were collected when available. The primary outcome was improvement in AIS defined as an improvement by at least one step on the AIS scale from admission to long-term follow-up. Another primary outcome, ambulatory status, was qualitatively measured and defined as the ability to walk with or without aid. Only patients dependent on a wheelchair for mobilization were categorized as wheelchair dependent. Patients who at times would use a wheelchair to simplify mobilization but were otherwise ambulatory with or without aids were classified as ambulatory. All wheelchair dependent patients required assistance for transfers to and from the wheelchair. The secondary outcomes were neuropathic pain, as subjectively reported by patients, and bowel and bladder dysfunction. Data on bladder and bowel function were recorded at regular assessments by a urotherapist and assistant nurse. Recorded data kept in the patient health record software include bowel emptying lists, anal sphincter function, the neurogenic bowel dysfunction (NBD) score, the Bristol stool scale, and the functional independence measure (FIM). In addition, studies such as abdominal CT and colonoscopy were performed when clinically indicated and kept in the electronic patient chart.

### Statistics

As all continuous data, including age, date from injury to admission, motor scores, days in the ICU, and follow-up times, deviated from a normal distribution pattern (Shapiro–Wilks test p-value < 0.05), we present them as median (interquartile range). Categorical data are presented as counts (percentages). McNemar’s and Wilcoxon signed-rank tests were used to assess status changes between admission and follow-up for binary and ordinal data, respectively. Univariable and step-down multivariable logistic regression analyses were used to determine predictors of improved AIS and ambulatory function. Patients who were AIS E on admission were excluded from the prediction of AIS improvement. Missing variables were handled via listwise deletion. R (version 4.1.2) was used for all analyses, and p < 0.05 was considered statistically significant.

## Results

### Baseline data

The study included 194 patients with a median age of 64 years, of whom 70% were male. Falls (65%) and traffic accidents (22%) were the predominant mechanisms of injury. Ninety percent of patients were admitted within 24 h of their SCI. Upon admission, the most frequent injury classifications were AIS C (35%), indicating an incomplete injury with some motor function preserved, and AIS D (32%), suggesting a muscle grade 3 or higher on at least half of the key muscles below the single neurological level of injury. Complete SCI, denoted as AIS A, was present in 24% of cases. At admission, 45 (23%) patients had a central cord syndrome. The median upper extremity motor score (UEMS) and lower extremity motor score (LEMS) were 18 and 15, respectively (Table 1). The most common level of injury was C5 (35%), followed by C4 (19%), and C6 (18%). A high signal intensity on T2 MRI of the spinal cord was found in 92%.

**Table 1:** Demographics and baseline data, treatment data and long-term outcomes.

Variable	All patients (n = 194)
Baseline data	
Age (years)	64 (46–73)
Male sex	136 (70%)
Injury mechanism	
Fall	126 (65%)
Traffic accident	43 (22%)
Assault	5 (2.6%)
Other	20 (10%)
Days from injury to admission	1.0 (1.0–1.0)
AIS on admission	
AIS A	46 (24%)
AIS B	19 (9.8%)
AIS C	67 (35%)
AIS D	62 (32%)
Central cord injury	45 (23%)
UEMS on admission	18 (9.0–28)
LEMS on admission	15 (0.0–35)
Highest level of injury on imaging	
C0–C1	10 (5.2%)
C2	18 (9.3%)
C3	28 (14%)
C4	37 (19%)
C5	61 (31%)
C6	34 (18%)
C7	6 (3.1%)
High signal on T2WI	169 (92%) (10 missing)
Treatment data	
Surgical treatment	183 (94%)
ICU treatment	113 (58%)
Days in ICU	8.0 (4.0–19)
Days in SCIU	37 (21–54) (1 missing)
Days in outpatient rehabilitation	42 (22–59) (3 missing)
Outcome data	
Follow-up time (years)	3.7 (1.3–6.0)
AIS improved	79 (41%)
Ambulatory	98 (51%)
Without walking aid	55 (28%)
With walking aid	43 (22%)

Data presented as median (interquartile range) or number (proportion).

*AIS* American spinal cord injury association impairment scale, *LEMS* lower extremity motor score, *T2WI* T2-weighted image, *UEMS* upper extremity motor score.

### Treatment and outcome

Surgical intervention was performed in 94% of patients. ICU was required in 58%, with a median stay of 8 days. The median SCIU-stay was 37 days (IQR 21–54 days), followed by a median of 42 days (OQR 22–59) spent at an outpatient rehabilitation clinic (Table 1).

**Fig. 1:**
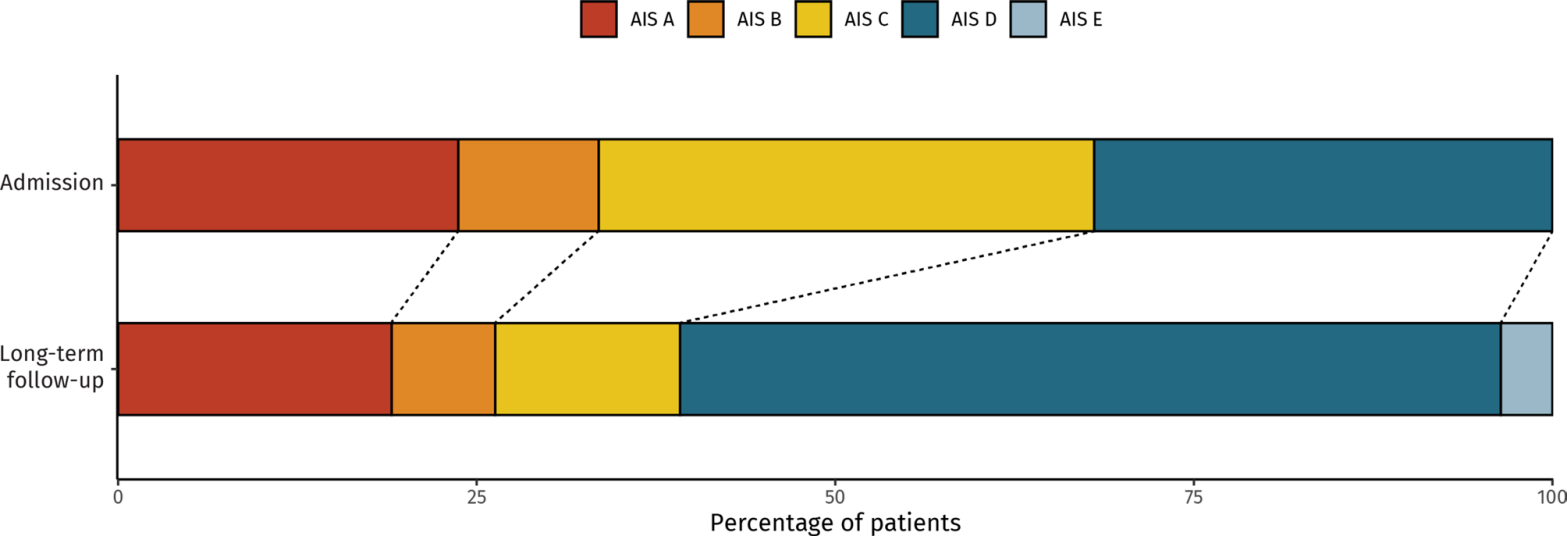
Stacked bar chart of relative proportion of patients categorized according to the ASIA impairment scale (AIS) on both admission and long-term follow-up.

The median follow-up time was 3.7 years (IQR 1.3–6.0 years). At this time, 41% (n = 79) had improved in AIS by at least one step, and 51% (n = 98) were ambulatory (Table 1). Of the ambulatory patients, 56% did not need a walking aid while 44% did. On comparing admission data with follow-up, a significant improvement in AIS (p < 0.001, Fig. 1) and reduction in patients with bladder and bowel dysfunction (65% to 53%, p < 0.001) was observed. Neuropathic pain showed no significant change (40% to 47%, p = 0.218) (Table 2). The median functional independence measure (FIM) total score at last follow-up, recorded in 168 of the 194 patients, was 94 (IQR 61–120).

**Table 2:** Comparison of AIS, pain and bladder and bowel function at admission and long-term follow-up.

Variable	Admission (n = 194)	Follow-up (n = 194)	p-value
AIS	–	–	** < 0.001**
AIS A	46 (24%)	37 (19%)	–
AIS B	19 (9.8%)	14 (7.2%)	–
AIS C	67 (35%)	25 (13%)	–
AIS D	62 (32%)	111 (57%)	–
AIS E	0 (0.0%)	7 (3.6%)	–
Neuropathic pain	78 (40%)	91 (47%)	0.218
Bladder & bowel dysfunction	127 (65%)	103 (53%)	** < 0.001**

Data presented as number (proportion).

*AIS* American spinal cord injury association impairment scale.

Bold text in the p-values column indicates a statistically significant association (p < 0.05). P-values shown are for paired testing.

Data from 3 to 5 years follow-ups were available in 64% (125/194) and 59% (114/194) of the cohort, respectively. At 3 years the distribution of AIS was A 21%, B 6%, C 14%, D 56% and E 2.5%. The proportion of ambulatory patients was 62/125 (50%) and of the ambulatory patients, 34/62 (55%) did not depend on a walking aid while 28/62 (45%) did. Half of the patients, 63/125 (50%) were wheelchair dependent. At 5 years follow up the distribution of AIS was A 23%, B 7.9%, C 12%, D 54% and E 3.5%. The proportion of ambulatory patients was 55/114 (48%), and of the ambulatory patients, 26/55 (47%) did not depend on a walking aid while 29/55 (53%) did. Roughly half of the patients, 59/114 (52%), were wheelchair dependent.

### Predictors of AIS improvement and ambulation

The multivariable analysis predicting at least a one-step improvement in AIS revealed several significant predictors. A higher likelihood of improvement was seen in patients with an initial AIS of C to D (OR 7.46, p < 0.001), central cord syndrome (OR 3.28, p = 0.016) and C0–C3 injury (OR 2.45, p = 0.017). Higher LEMS (OR 0.92, p < 0.001) and longer ICU-stay (OR 0.96, p = 0.011) were negative prognostic factors (Table 3).

**Table 3:** Predictors of improved AIS at long term follow-up.

Variable	Univariable model		Step-down multivariable model	
	OR (95% CI)	p-value	OR (95% CI)	p-value
Age (years)	1.01 (0.99–1.02)	0.272	–	–
Male sex	1.07 (0.57–2.01)	0.883	–	–
C0–C3 injury	2.10 (1.12–3.96)	**0.021**	2.45 (1.19–5.17)	**0.017**
AIS C–D on admission	1.90 (1.02–3.61)	**0.047**	7.46 (2.73–21.4)	** < 0.001**
Days from injury to admission	0.98 (0.89–1.01)	0.500	–	–
UEMS on admission	0.97 (0.95–0.99)	**0.005**	–	–
LEMS on admission	0.99 (0.97–1.00)	0.108	0.92 (0.88–0.95)	** < 0.001**
Central cord injury	1.74 (0.89–3.42)	0.108	3.28 (1.29–9.05)	**0.016**
High signal on T2WI	0.83 (0.28–2.46)	0.727	–	–
Surgery required	0.81 (0.24–2.92)	0.743	–	–
ICU-stay (days)	0.98 (0.95–1.00)	0.061	0.96 (0.92–0.99)	**0.011**

*AIS* American spinal cord injury association impairment scale, ICU intensive care unit, *T2WI* T2-weighted image.

Bold text in the p-values column indicates a statistically significant association (p < 0.05).

The multivariable analysis predicting long-term ambulatory function showed a higher likelihood of ambulation in patients with an initial AIS of C to D (OR 49.9, p < 0.001) and higher LEMS (OR 1.09, p < 0.001), while older age was a negative prognostic factor (p = 0.003, Table 4).

**Table 4:** Predictors of ambulatory function at long-term follow-up.

Variable	Univariable model		Multivariable model	
	OR (95% CI)	p-value	OR (95% CI)	p-value
Age (years)	1.01 (0.99–1.02)	0.439	0.95 (0.92–0.98)	**0.003**
Male sex	0.63 (0.33–1.16)	0.142	–	–
C0–C3 injury	0.97 (0.52–1.81)	0.927	–	–
AIS C–D on admission	91.6 (26.6–578)	** < 0.001**	49.9 (9.91–411)	** < 0.001**
Days to admission	1.16 (1.01–1.45)	0.106	–	–
UEMS on admission	1.09 (1.06–1.12)	** < 0.001**	–	–
LEMS on admission	1.13 (1.09–1.16)	** < 0.001**	1.09 (1.05–1.13)	** < 0.001**
Central cord injury	6.67 (3.04–16.3)	** < 0.001**	–	–
High signal on T2WI	0.95 (0.32–2.76)	0.925	–	–
Surgery required	1.24 (0.36–4.44)	0.730	–	–
ICU-stay (days)	0.89 (0.84–0.93)	** < 0.001**	–	–

*AIS* American spinal cord injury association impairment scale, *ICU* intensive care unit, *T2WI* T2-weighted image.

Bold text in the p-values column indicates a statistically significant association (p < 0.05).

## Discussion

In this population-based cohort study, we examined the long-term outcomes and determinants of neurological recovery in patients with cervical SCI. Most injuries were incomplete, and almost all patients underwent surgery. A large proportion of patients improved in AIS grade at long term follow up and the initial injury severity was identified as a predictor of long-term outcome. At follow up, half of the patients were ambulatory. While AIS and bladder and bowl function improved, neuropathic pain remained unchanged. Older age was a negative prognostic factor. With a median age of 64 years, the cohort reflects the trend towards an aging population being affected by cervical SCI. This highlights the importance of geriatric care and may signal a need for more robust fall prevention strategies in this demographic.

The admission data revealed that most injuries resulted in incomplete SCI, with 35% classified as AIS C and 32% as AIS D. The preservation of motor functions suggests a potential for recovery and predicts benefits of rehabilitation. The finding of complete SCI (AIS A) in nearly a quarter of the patients, indicates a group for whom recovery is much more challenging and where gains in rehabilitation may require a greater effort. Complete neurological recovery (AIS E) was not achieved in any of the patients initially graded as AIS A, B, or C (Fig. 1). However, many of them witnessed neurological improvements, which underlines the necessity for sustained rehabilitation efforts. For example, around 20% of individuals who were AIS A improved by at least one step, consistent with a prior meta-analysis reporting that 19% (95% CI 16.2–22.6%) transitioned from complete to incomplete injury^13^.

While there was a significant reduction in bladder and bowel dysfunction, neuropathic pain showed no improvement with rehabilitation. Neuropathic pain may be debilitating and have a great negative impact on the individual’s quality of life^14,15^ and rehabilitation efforts^16,17^. Strategies to effectively manage neuropathic pain are important to achieve the best outcomes.

### Predicting AIS improvement

Patients with less severe injuries on admission (AIS C–D) exhibited a significantly greater chance of improvement. This illustrates the importance of the severity of the initial injury in defining the recovery potential, a finding consistent with the existing literature^13,18^.

Central cord syndrome describes an incomplete injury to the cervical spinal cord, selectively affecting the more sensitive central grey matter structures that govern motor function in the hands and arms, while sparing lower extremity function. Central cord syndrome was associated with an increased likelihood of AIS improvement, in line with published data^19^.

Our analysis indicated that a longer stay in the ICU was associated with a decreased likelihood of AIS improvement. An extended ICU stay may indicate a more severe injury with a greater risk for complications, which may lead to a less favorable prognosis.

An association between a higher level of injury (C0–C3) and improved AIS was seen. This finding is counterintuitive, as higher injuries typically correlate with poorer outcomes due to the greater extent of neurological compromise.

Despite being extensively studied, the recovery after spinal cord injury remains unpredictable due to the heterogeneity of the condition. For instance, granular data is lacking regarding the difference in outcomes of upper and lower cervical SCI^20^. Available studies suggest that severe neurological impairment and older age are linked to limited neurological recovery.

Surprisingly, our analysis showed that an injury at C0-C3 level was associated with a long-term improvement in AIS score. As this goes contrary to our current understanding of spinal cord injury and recovery mechanisms, we suggest that it may be the effect of a sampling bias. Since the more severe upper cervical injuries are fatal, patients with milder injuries and a greater potential for neurological recovery are more likely to survive.

In a recent prospective study with data from 470 patients with cervical SCI, Futch et al. concluded that there were group-wise differences between upper and lower cervical SCI^20^. Upper cervical SCI were associated with diabetes and falls and had a better AIS score at presentation (AIS C), while lower injuries were associated with sports, had a greater frequency of complications, and presented with worse AIS score (AIS A).

The dimensions of the cervical spinal canal are related to sex, age, height and spinal level and the spinal canal is narrower at C6 than at C3 where it widens gradually towards C1 and the foramen magnum^21^. Hence, the lower cervical spinal cord is anatomically more susceptible to injuries decreasing the width of the spinal canal.

In summary, patients with less severe status at admission, particularly those with central cord syndrome, showed a higher likelihood for improvement in AIS scores, while extended ICU stays correlated with less favorable outcomes, potentially due to more severe injuries or complications arising from prolonged intensive care.

### Predicting ambulatory function

Fifty-one percent of the patients were ambulatory at follow-up, with 22% using walking aids. The increased likelihood of regaining ambulatory function in patients with initial AIS scores of C to D and greater LEMS can be explained by the residual motor function these patients retain. The preserved function suggests partial preservation of the spinal cord pathways, which can be rehabilitated over time, leading to improved outcomes in walking ability. This is consistent with evidence in the literature, which shows that patients with complete injury have a low chance of regaining ambulatory function^22,23^. The finding that old age was a negative prognostic factor may reflect a reduced recovery potential relating to senescence itself as well as to a greater degree of pre-existing comorbidities. Elderly patients often present a greater challenge to healthcare providers. The vulnerability, or frailty, of elderly individuals, is often discussed in the literature^24^. Older individuals may have comorbidities that interfere with and prolong the rehabilitation process^25^. A prolonged length of hospital stay may also be necessary due to a greater susceptibility to and slower recovery from secondary complications^26^. In addition, a more thorough and complex planning may be needed to ensure adequate follow up after discharge.

Tailored rehabilitative strategies, such as extended inpatient rehabilitation periods, and multidisciplinary and multi-specialty management ought to be explored. Specialized rehabilitation units for older individuals with SCI, may be advantageous for this patient group and may lead to an improvement in functional and neurological outcomes.

### Bowel and bladder dysfunction

Most individuals with SCI, both complete and incomplete, experience neurogenic bladder and bowel dysfunction with a negative impact on quality of life^27,28^. Adriaansen et al. reported that up to 81% of individuals with SCI presented with a varying degree of bladder dysfunction^29^. Similarly, Pavese et al. reported that up to 80% of individuals with SCI had bowel dysfunction^30^. A longitudinal study on a population of individuals with SCI for at least 20 years, reported that by the 6 year follow up the method of bladder emptying had been changed in 29% of the cases^31^. Bowel and bladder dysfunction is also common in vascular SCI caused by spinal cord infarction^32–34^. Thus, long-term support is needed from health care providers to ensure an adequate management of bladder and bowel dysfunction after SCI.

In the present cohort, a reduction in bladder and bowel dysfunction was documented, with a 12% shift from 127 patients (65%) at admission down to 103 (53%) at follow-up. Even though the 12% reduction implies a trend towards improved outcomes, more than half of the cohort still suffers bladder and bowel dysfunction at long-term outcome, a fact highlighting the magnitude of the condition and the need for continued efforts to improve management and research new treatments.

### Neuropathic pain

Neuropathic pain is common in the aftermath of a SCI, usually presenting as a chronic condition that is difficult to treat and is reported in 60–69% of individuals with SCI^14,35^. Previous studies reported a positive correlation between advanced age at the time of injury and the prevalence of neuropathic pain^36^. In our cohort, with a median age of 64 years, neuropathic pain occurred in 47% at follow-up. Similarly, a Swedish study on 456 individuals with SCI reported neuropathic pain in 45.7%^37^, and a meta-analysis on the prevalence of neuropathic pain after SCI reported a pooled prevalence of 53% and a greater frequency in older tetraplegic individuals^38^. Currently, the available treatment arsenal include pain relief alternatives which have often been regarded as suboptimal^39^. In combination with the fact that neuropathic pain affects approximately half of the SCI population, the poor efficacy of available treatments underscores the need for research to enhance treatment options and efficacies.

### Strengths and limitations

This study’s strength lies in its comprehensive, population-based approach, analyzing a decade’s worth of data from a single, specialized care unit. The large sample size and the long-term follow-up provide a robust dataset for evaluating outcomes. To make best use of the available data, the choice was made to compare admission data to last available long-term follow up. The median of which was at 3.7 years. The alternative, to analyze data at fixed time points would have weakened the analysis due to many missing data points. The reason for this is that clinical follow ups were scheduled in relation to the need of the individual patient, which became more and more diverse as time went by. However, available AIS and ambulation data at 3- and 5 years follow-ups, when available, did not differ from the compound long-term follow up data. Another strength of this study is its setting in Stockholm, Sweden, where access to state-funded healthcare eliminates the variability in care due to patients’ financial limitations. This uniformity allows for a more accurate assessment of cervical SCI outcomes, unencumbered by the disparities often seen in healthcare systems where treatment access and quality are influenced by personal resources.

The retrospective nature of the data restricts the ability to establish causality. The study’s confinement to a single institution, while allowing for consistent treatment protocols, raises questions about the broader applicability of the findings across different healthcare systems with varying practices and patient populations. In adherence to the institutional guidelines, SCI AIS were not routinely evaluated with neurophysiological studies. Thus, the clinical diagnosis of a complete SCI, AIS A, does not preclude the existence of fibers traversing the injury and able to convey corticospinal signals. Data on bladder and bowel function were not granular enough to reliably differentiate between the relative contribution of these two aspects at the individual level. Thus, the data reflects a dichotomy of whether a normal function exists or not. Lastly, while the study encompassed a range of clinical variables, unmeasured factors like psychosocial support, patient resilience, and specific rehabilitation protocols may impact the recovery after cervical SCI.

### Conclusion

This study highlights the importance of the initial injury severity for the long-term prognosis in cervical SCI, while it simultaneously reveals the recovery potential that exists even in severe cases. It underscores the critical role that individualized rehabilitation efforts play in supporting meaningful recovery and in improving patient outcomes.

## Data Availability

Data that support the findings of this study are available on request from the corresponding author, AET.
